# Current trends in telemedicine for chronic disease management

**DOI:** 10.6026/973206300210587

**Published:** 2025-04-30

**Authors:** Navaneeth Ranjith, Sree Nithish Kanna Baskaran, Saranya R., Aswin Ganesan, Ranjani Kanakaraj, Gajalakshmi S., Siraj Muhammad Shafy, Akshay Sureshkumar Nair

**Affiliations:** 1 Department of Urology, Caritas Hospital, Kottayam, India; 2 Duty Medical Officer, ICU Department, SRM Global Hospitals, Kattankulathur, Tamil Nadu, India; 3 Department of Community Medicine, Madha Medical College and Research Institute, Chennai, India; 4 Department of Paediatrics, Madras Medical College, Chennai, India; 5 Institute of Internal Medicine, Madras Medical College, Chennai, India; 6 Department of Community Medicine, Veer Chandra Singh Garhwali Government Institute of Medical Science and Research, HNB Teaching & Base Hospital, Srinagar, Pauri Garhwal, Uttarakhand, India; 7 Department of Neurosurgery, VPS Lakeshore Hospital, Kochi, Kerala, India; 8 Department of General Medicine, Starcare Hospital, Calicut, India

**Keywords:** Telemedicine, chronic disease management, bibliometric analysis, global research trends, healthcare accessibility, telehealth innovation, research impact

## Abstract

Telemedicine is becoming a transformative tool for managing chronic diseases. Known literature data emphasizes the effectiveness and accessibility of telemedicine and its impact on patient outcomes. Thus, the foundation for advancing the role of telemedicine in chronic disease management for improving healthcare accessibility and quality of life is imperative.

## Background:

The revolutionary tool of chronic disease management is telemedicine, offering multiple solutions to enhance patient outcomes optimize resource use and eventually improve health care. Telemedicine adoption will maintain continued distance monitoring of patients, ensuring timely interventions and also provide better access to health care services for patients with diabetes, hypertension and cardiovascular diseases [1]. This will benefit the patients staying in rural areas or in less provision of health care services. Since chronic diseases are on the increase all over the world, telemedicine has emerged with great emphasis as a tool that may serve as a potential means to ease off the burden on the health system and provide more affordable solutions to care with time [2]. Chronic diseases represent, nowadays, the major cause of morbidity and mortality in the world, so new management approaches are evolving. In this respect, through regular monitoring performed in a timely and careful manner, telemedicine ensures better control over the disease, its better compliance with the treatment plan and also a reduced rate of readmissions to the hospital [3].

The past decade has, indeed experienced an explosion of studies dealing with the application of telemedicine in chronic diseases management. Several studies highlighted that such telemedicine effects had an imperative and key role in lowering health care costs, ensuring quality of care and increasing the satisfaction of patients [4]. In addition, the COVID-19 epidemic inspired interest in adopting telemedicine and its ability to ensure the continuation of care during such a health crisis [5]. Management of chronic diseases through telemedicine is also much diversified and complex and comprises numerous disciplines, technologies and methodologies. The amount of diversity was vast, and a multi-dimensional analysis required assessing trends, impact and direction of the research in this area. This brings in importance a bibliometric analysis that measures trends for the publication, co-authorship networks and citation classics of the research into the area of telemedicine [6]. Analysis in this regard would be beneficial to the researchers, policymakers and healthcare providers optimize telemedicine strategies for chronic disease care [7]. It is a bibliometric analysis of global trends and the impact of telemedicine in the management of chronic diseases with emphasis on some key thematic research issues, influential authors and also patterns of collaboration. These areas shall give an overall view that contributes to future research developments and policies in telemedicine [8]. Therefore, it is of interest to review the current trends in telemedicine for chronic disease management.

## Methodology:

This is a bibliometric analysis meant to quantify global research trends and further establish how telemedicine impacts chronic diseases. In so doing, the methodology used involves a systematic approach both in terms of data collection as well as methodology and search strategy used to ensure it is extensively thorough and accurate enough to demonstrate the literature within the realm.

## Data source:

The data for this paper were retrieved from PubMed, which is a very credible database for biomedical literature. PubMed is particularly known for its outstanding coverage and accurate indexing of articles about medicine and health; therefore, it is a suitable place for doing a study like this bibliometric analysis.

## Search strategy:

A general search strategy for this study was formulated on telemedicine and the applications including chronic disease management.

## The search terms used included:

Telemedicine-related terms: "Telemedicine" [MeSH], "Telehealth", "Remote Consultation," "eHealth" and "mHealth." Chronic disease-related terms: "Chronic Disease" [MeSH], "Chronic Illness", "Disease Management" [MeSH] and "Long-term Care."

## The final search query used was:

The last search term used in the search is below: ("Telemedicine" [MeSH] OR "Telehealth" OR "Remote Consultation" OR "eHealth" OR "mHealth") AND ("Chronic Disease" [MeSH] OR "Chronic Illness" OR "Disease Management" [MeSH] OR "Long-term Care"). This search was narrowed to studies published in the last 20 years between 2004 and 2024, which limited the period to only current trends and telemedicine-related developments. Such a time factor has created the possibility of selecting present research improvement and recent trend developed over the last two decades.

## Data extraction:

From 3,955 search results, there were different varieties of studies associated with the application of telemedicine in chronic disease management. Each record retrieved was scrutinized for the key bibliometric information of title, authors, affiliations, journal name and year of publication, keywords and number of citations. The abstract and type of the publication for each article were also verified Check to ensure whether the chosen article is pertinent to the topic under study. Only those articles that were related to the utilization of telemedicine in the care of chronic diseases were included for analysis. All other unrelated publications were excluded.

## Bibliometric analysis tool:

Bibliometric analysis was performed using Biblioshiny in R Studio. It is a web application and part of the bibliometrix R package. Consequently, it has been applied in conducting extensive and multifaceted types of bibliometric analysis.

## The analysis comprised:

## Annual publication trends:

Analyze the year-on-year trend in the growth of the annual period of publications so that it may be seen whether or not such trends are in telemedicine research on chronic disease management.

## Authorship and institutional analysis:

Identify who the most productive authors and institutions were in contributing to the field.

## Journal analysis:

Determine the most important journals publishing on telemedicine and chronic disease as an attempt to find out which publications are most frequently used.

## Citation analysis:

Number of highly cited articles to identify influential studies that would have shaped the present research landscape.

## Collaboration networks:

Global networks of author and institution collaboration, to draw networking and regional contributions.

## Data analysis and visualization:

Bibliophagy supported the descriptive and inferential analysis of the bibliometrics in this research. The package made easier the drawing of publication, citation and co-authorship networks through tables and tables that described influential topics of research, geographic distribution and collaborative efforts.

## Coverage:

This bibliometric analysis will cover studies on telemedicine and chronic disease within the last two decades. The dataset encompasses 3,954 documents extracted from 1,113 sources by more than 19,000 authors. Major metrics include annual growth in publications of 21.05%, average co-authors per document being 6.55 and average document age being 7.23 years. The main characteristics of the dataset, including documents, authors and percentage of international co-authorship, are summarized in Table 1.

## Results and Discussion:

The increasing research interest in telemedicine's applications for chronic disease management is known.

Table 2 illustrates the production of major affiliations over time, with significant growth in output from 2015 onwards. Trends in Publications by Affiliation - Leading institutions have contributed extensively to telemedicine research. The most prolific institutions include the University of Toronto, University of Washington and Radboud University Medical Center, each producing substantial research output over the years. Table 3 highlights the most relevant affiliations based on article count, demonstrating the University of Toronto's leading role in this field (Table 3). Geographical Distribution of Publications - The United States, China, Canada and Australia are among the leading contributors to telemedicine research, showing substantial growth over the last decade. This distribution reflects the global focus on using telemedicine for managing chronic diseases. Table 4 presents the cumulative growth of research output by country, with the USA demonstrating the most significant increase. Table 5 provides a geographical map of scientific production, indicating high research activity in North America, Europe and parts of Asia and Oceania. International collaboration has been an essential aspect of telemedicine research. Table 6 illustrates the global collaboration network, showing active research partnerships between countries, with the USA, China and European nations playing central roles. The most prolific authors in telemedicine for chronic disease management include researchers such as Li, Chavannes NH and Sheikh A. Their work has been highly influential in advancing research in this field (Table 7). Lists the most relevant authors based on the number of publications. These key contributors have shaped the research landscape, highlighting the importance of individual researchers in this evolving field. Regarding influential journals, the journal of medical internet research, telemedicine journal and e-Health and Journal of Telemedicine and Telecare are the most prominent sources for telemedicine studies. Table 8 shows the production trends of these sources over time. Table 9 lists the top journals by document count. Research Themes and Topics: Telemedicine research has evolved over the years to cover various themes, such as digital health, patient satisfaction, mobile applications and remote monitoring. Table 10 displays the trending topics within telemedicine research, with terms like "telemedicine", "chronic disease" and "quality of life" being frequently discussed in recent years. This trend analysis reveals the expanding scope of telemedicine applications for chronic disease care. Keyword co-occurrence analysis further explores relationships among frequently used terms in telemedicine research. Table 11 provide co-occurrence networks that visualize these connections, showing clusters around core terms such as "humans", "telemedicine" and "chronic disease." Provide a visual overview of commonly used terms, a word cloud in highlights terms like "humans", "telemedicine" and "chronic disease" as central to the research on telemedicine for chronic illness management. Finally, an analysis of author productivity reveals compliance with Lotka's Law, indicating a few authors contribute a majority of the research output, while many authors contribute less frequently. Table 12 illustrates author productivity, confirming that a small percentage of authors have published extensively on telemedicine for chronic disease management.

The finding of this bibliometric study points out that telemedicine is important in chronic disease care, which has been rapidly increasing in the past two decades. This growth resonates with an international response to the increasing burden of chronic diseases and the increasing demand for new models for healthcare delivery [9]. Telemedicine has unique benefits for the management of chronic disease by allowing remote monitoring, early intervention and easy access to healthcare services [10]. Global Trends and Growth in Telemedicine Research Publication growth in telemedicine has been extremely rapid, especially since 2015. Such a trend is indeed consistent with other global health initiatives aimed at driving digital health as an applicable approach to chronic disease management [11]. The COVID-19 pandemic accelerated this trend even further, considering that worldwide healthcare systems had to shift towards remote care solutions. Also related studies during the research for digital health indicate similar trends in publications provoked by technological changes as well as to reduce direct physical contact in the health sector [12]. This is supported by the current analysis: the annual growth rate amounts to 21.05% in telemedicine-related publications. Geographic and Institutional Contributions had the most major contributions from institutions located in North America, Europe and parts of Asia. Distinct types of institutions in these regions head the publication counts spread across the USA, China and Canada. This is a pattern consistent with earlier literature, where such countries have made enormous investments in telemedicine infrastructure and research, most probably because of the high prevalence of chronic diseases and an aging population [13]. Such an international collaboration network also represents the international nature of telemedicine research, such as collaborations that cross more than one country. International collaborations help in the use and sharing of both resources and expertise as well as best practices both for the implementation and research in telemedicine. Some of the key authors include Li and Chavannes, who made significant contributions to the body of literature that stressed the role of individual researchers in advancing telemedicine research. New findings will also be published in several influential journals, which include Journal of Medical Internet Research and Telemedicine Journal and e-Health. These outlets remain at the forefront in publishing high-impact studies that dictate the course of telemedicine research [14]. In this case, the concentration of influential journals in these outlets underlines their importance in terms of audience reach and how they influence health policy and practice. Keyword co-occurrence and trend analysis show topics like "digital health, patient satisfaction" and "remote monitoring" has gained relevance over the years. This shift indicates that there is now an explicated scope of research, which not only encompasses technology but also the outcome of the patients, who play a vital role in chronic disease management. Increasing terms such as "quality of life" and "self-management" are also indicative of the rising interest in holistic care models based on patient empowerment and involvement [15]. Furthermore, the trend topics suggest that telemedicine is flexible to chronic conditions such as cardiovascular diseases, diabetes and chronic respiratory diseases. Such results are in consonance with previous literature which reported telemedicine is suitable for addressing management requirements of such conditions through continuous surveillance and appropriate timely interventions [16]. Although this bibliometric review offers useful insights, a few limitations of this study need to be mentioned. Dependency on PubMed as the only search source might lead to missing highly relevant studies indexed in other databases, Scopus or IEEE Xplore. Search in several databases may give a better overall impression of the research conducted on telemedicine. Though the analysis of co-occurrence and trend provides a summary idea about research themes, deeper qualitative analyses are suggested to understand such studies' context and nuances [17]. Future Research in Telemedicine must then involve further investigation of artificial intelligence and machine learning in predictive analytics for chronic disease management [18]. Telemedicine dependency will also raise a number of issues about security on the data that it deals with, patient confidentiality and regulation compliance-all of which will impact on the re-acceptance of patients and honest practice [19]. Research currently focuses on developing a comprehensive e-Health framework, optimizing m-Health for chronic diseases, improving e-Health intervention trial reporting, assessing social factors in e-Health literacy and examining telemedicine's role during the Covid-19 pandemic [20]. Existing studies reflect the heterogeneity in study design, population and outcome measures. There is a need for further studies on the effects of digital interventions on related health literacy for individuals with chronic conditions [21]. The application of telemedicine is well established across countries in the WHO European Region; however, some countries could still benefit from the many uses of these digital solutions [22]. As shown by different studies as Artificial Intelligence for e-Health [23]. Finally, there is a need for additional research to produce the long-term impacts of telemedicine in healthcare relating to cost-effectiveness, especially in low-resource regions where more people are getting chronic diseases. Though this is not the largest scale of bibliometric research concerning telemedicine [11], we revealed the changing patterns of top ranking research areas while others did not, among those bibliometric researches in topics relevant to [24].

## Conclusion:

The growing role of telemedicine in chronic disease care is evident. It reflects the global trend toward a change in health care delivery into its digital solutions, driven by technological advancement with a need to be accessible and cost-effective. Thus, optimization of these technologies for diverse healthcare systems will be needed along with conquering ethical concerns, ensuring that all people around the globe have equal access to telemedicine services.

## Figures and Tables

**Table 1:** Overview of data characteristics in telemedicine research on chronic disease management

**Metric**	**Value**
Timespan	1994-2024
Total Documents	3,954
Total Sources	1,113
Authors	19,753
Annual Growth Rate	21.05%
Co-Authors per Document	6.55
Average Document Age	7.23
International Co-Authorship	13.08%

**Table 2:** Annual scientific production over time

**Year**	**Number of Articles**
2024	308
2023	243
2022	270
2021	350
2020	465
2019	291
2018	245
2017	238
2016	225
2015	216

**Table 3:** Affiliations' production over time

**Affiliation**	**Year**	**Articles**
University of Toronto	2024	338
Mcmaster University	2024	115
University of Washington	2024	190
University Health Network	2024	123
Radboud University Medical Center	2024	132
University of Toronto	2023	314
Mcmaster University	2023	105
University of Washington	2023	176
University Health Network	2023	114
Radboud University Medical Center	2023	121
University of Toronto	2021	258
Mcmaster University	2021	71
University of Washington	2021	102
University Health Network	2021	99
Radboud University Medical Center	2021	81
University of Toronto	2020	207
Mcmaster University	2020	58
University of Washington	2020	93
University Health Network	2020	61
Radboud University Medical Center	2020	66

**Table 4:** Most relevant affiliations in telemedicine research

**Affiliation**	**Articles**
University of Toronto	338
University of Washington	190
Radboud University Medical Center	132
University Health Network	123
Mcmaster University	115
The University of Sydney	111
University of Oxford	109
Monash University	104
The University of Queensland	103
University of Alberta	102
University of California	102

**Table 5:** Country production over time

**Country**	**Year**	**Articles**
USA	2024	3965
AUSTRALIA	2024	1961
CANADA	2024	1698
CHINA	2024	1342
NETHERLANDS	2024	1082
USA	2023	3496
AUSTRALIA	2023	1760
CANADA	2023	1458
CHINA	2023	1154
NETHERLANDS	2023	940
USA	2022	3006
AUSTRALIA	2022	1575
CANADA	2022	1266
CHINA	2022	1026
NETHERLANDS	2022	826
USA	2021	2512
AUSTRALIA	2021	1368
CANADA	2021	1169
CHINA	2021	683
NETHERLANDS	2021	730

**Table 6:** Country scientific production map (darker blue shows larger production)

**Region**	**Freq**
USA	3965
AUSTRALIA	1961
CANADA	1698
CHINA	1342
NETHERLANDS	1082
ITALY	1078
SPAIN	955
GERMANY	639
FRANCE	476
UK	444
NORWAY	426
SWEDEN	410
DENMARK	409
SWITZERLAND	244

**Table 7:** Country collaboration map

**USA**	**CHINA**	**30**
ITALY	SPAIN	28
NETHERLANDS	BELGIUM	27
USA	NETHERLANDS	27
NETHERLANDS	GERMANY	26
USA	CANADA	26
NETHERLANDS	ITALY	25
SPAIN	GERMANY	25
USA	AUSTRALIA	22
NETHERLANDS	SPAIN	21
ITALY	GERMANY	20
USA	ITALY	20

**Table 8:** Most relevant authors in telemedicine research

**Authors**	**Articles**	**Fractionalised Articles**
Li Y	26	2.48834445
Chavannes NH	22	4.04675668
Sheikh A	21	3.49902557
Mckinstry B	20	5.65634921
Scalvini S	19	3.20400433
Li J	18	2.68621934
Kroenke K	17	4.24563492
Wang Y	17	2.527351
Wang X	16	1.79361224
Cafazzo JA	15	3.83730159
Campbell KI	15	2.36287185

**Table 9:** Sources' production over time

**Year**	**Journal of medical internet research**	**Telemedicine journal and e-health: the official journal of the american telemedicine association**	**Journal of elemedicine and telecare**	**Studies in health technology and informatics**	**Jmir M health and health**
2024	202	175	165	153	103
2023	183	162	154	143	93
2022	165	152	148	139	85
2021	141	146	145	134	78
2020	123	143	140	130	67
2019	91	138	130	124	39
2018	75	126	122	119	6
2017	67	117	113	111	4
2016	52	109	97	100	2
2015	41	100	93	91	1
2014	26	89	84	67	1

**Table 10:** Most relevant sources in telemedicine research

**Sources**	**Articles**
Journal of medical internet research	202
Telemedicine journal And E-Health: The official journal of the american telemedicine association	175
Journal of telemedicine and telecare	165
Studies in health technology and informatics	153
Jmir Mhealth and Uhealth	103
Bmj open	54
International journal of environmental research and public health	51
International journal of medical informatics	50
International journal of chronic obstructive pulmonary disease	48
Bmc health services research	47
Plos one	43
Annual international conference of the ieee engineering in medicine and biology society. Ieee engineering in medicine and biology society. annual international conference	31
Bmc medical informatics and decision making	31

**Table 11:** Trend topics in telemedicine and chronic disease management

**Term**	**Frequency**	**Year (Q1)**	**Year (Median)**	**Year (Q3)**
Humans	3778	2014	2018	2021
Female	1493	2015	2018	2020
Male	1440	2014	2018	2020
Telemedicine	1209	2015	2020	2022
Chronic disease	910	2012	2019	2022
Disease management	810	2011	2016	2020
Adult	780	2015	2019	2021
Telemedicine/methods	648	2015	2017	2020
Quality of life	441	2016	2019	2022
Aged, 80 and over	387	2013	2017	2019
Treatment outcome	319	2013	2016	2019
Chronic disease/therapy	314	2012	2016	2019
United states	280	2007	2013	2019
Sars-cov-2	276	2020	2021	2022

**Table 12:** Keyword co-occurrence network

**Node**	**Cluster**	**Betweenness**	**Closeness**	**Page Rank**
Humans	3	47.2459583	0.02083333	0.12301448
Telemedicine	3	4.01888231	0.01960784	0.04219075
Chronic disease	3	2.82253787	0.02040816	0.03299716
Disease management	3	1.98498571	0.02040816	0.03105883
Chronic disease/therapy	3	0.3787592	0.02	0.01399896
United states	3	0.1792429	0.02083333	0.01210075
Mobile applications	3	0.18976716	0.02040816	0.01111064
Child	3	0.07680416	0.01923077	0.01055849
Telemedicine/organization & administration	3	0.06863488	0.01851852	0.00841237
Retrospective studies	3	0.06276918	0.01851852	0.01000107
